# Genome-Wide Identification of the PHD-Finger Family Genes and Their Responses to Environmental Stresses in *Oryza sativa* L.

**DOI:** 10.3390/ijms18092005

**Published:** 2017-09-19

**Authors:** Mingzhe Sun, Bowei Jia, Junkai Yang, Na Cui, Yanming Zhu, Xiaoli Sun

**Affiliations:** 1 Key Laboratory of Agricultural Biological Functional Gene, College of Life Science, Northeast Agricultural University, Harbin 150038, China; kaik127@163.com (M.S.); jiabowei_paper@163.com (B.J.); 2 Crop Stress Molecular Biology Laboratory, College of Agronomy, Heilongjiang Bayi Agricultural University, Daqing 163319, China; yjkfzsw@163.com; 3 LGPM, CentraleSupélec, Université Paris-Saclay, SFR Condorcet FR CNRS 3417, Centre Européen de Biotechnologie et de Bioéconomie (CEBB), 3 rue des Rouges Terres, 51110 Pomacle, France; na.cui@centralesupelec.fr

**Keywords:** *Oryza sativa*, PHD-finger transcription factors, evolutionary divergence, expression analysis, environmental stress

## Abstract

The PHD-finger family has been demonstrated to be involved in regulating plant growth and development. However, little information is given for its role in environmental stress responses. Here, we identified a total of 59 PHD family genes in the rice genome. These OsPHDs genes were located on eleven chromosomes and synteny analysis only revealed nine duplicated pairs within the rice PHD family. Phylogenetic analysis of all OsPHDs and PHDs from other species revealed that they could be grouped into two major clusters. Furthermore, OsPHDs were clustered into eight groups and members from different groups displayed a great divergence in terms of gene structure, functional domains and conserved motifs. We also found that with the exception of *OsPHD6*, all OsPHDs were expressed in at least one of the ten tested tissues and OsPHDs from certain groups were expressed in specific tissues. Moreover, our results also uncovered differential responses of OsPHDs expression to environmental stresses, including ABA (abscisic acid), water deficit, cold and high Cd. By using quantitative real-time PCR, we further confirmed the differential expression of OsPHDs under these stresses. *OsPHD1*/*7*/*8*/*13*/*33* were differentially expressed under water deficit and Cd stresses, while *OsPHD5*/*17* showed altered expression under water deficit and cold stresses. Moreover, *OsPHD3*/*44*/*28* displayed differential expression under ABA and Cd stresses. In conclusion, our results provide valuable information on the rice PHD family in plant responses to environmental stress, which will be helpful for further characterizing their biological roles in responding to environmental stresses.

## 1. Introduction

Rice (*Oryza sativa* L.) is one of the staple crops, feeding more than half of the world’s population. However, rice yields and quality are severely limited by diverse environmental stresses, especially cold, water deficit and high cadmium (Cd) [1]. Rice originates in tropical and subtropical regions, so it is much more sensitive to cold stress, compared with other cereal crops. Therefore, rice production, especially in high latitude regions, is severely prohibited by cold stress [2]. Another constraint comes from water deficit, particularly in arid and semi-arid regions [3]. Sustainable water is required during rice cultivation, hence rice is extremely sensitive to water deficit. Besides, due to excessive application of phosphate fertilizers during recent years, Cd concentration in soils has dramatically increased, which has become another limiting factor to rice productivity and quality [4]. Environmental stresses, especially cold, water deficit and high Cd, impose a broad range of restrictions on rice production.

To cope with these adverse environmental stresses, the expression of a considerable amount of genes is altered and a series of complex signal pathways is triggered in rice [5]. It has been suggested that transcription factors, including the well-known WRKYs [6,7], MYBs [8], NACs [9,10] and bZIPs [11], exert important roles in stress signal transduction. Among them, the zinc-finger transcription factors are widely distributed in plants and make crucial contributions to plant stress tolerance.

The zinc-finger proteins are characterized by a zinc-binding finger domain comprising of cysteines and/or histidines [12]. The conserved cysteine and histidine residues, in conjunction with zinc ions, can stabilize the spatial structure of zinc-finger proteins. According to the arrangement of the zinc-binding residues, zinc-finger proteins are classified into different types, including RING (Really Interesting New Genes), LIM (Lin11, Isl-1 and Mec-3) [13] and PHD (plant homeodomain) [14].

The PHD-finger domain is comprised of approximately 60 amino acids, with a characteristic feature Cys4–His–Cys3 (C-X_2_-C-X_(8–25)_-C-X_(2–4)_-C-X_(4–5)_-H-X_2_-C-X_(12–32)_-C-X_(2–3)_-C), which is similar to RING (Cys3–His–Cys4) and LIM (Cys2–His–Cys5) [15,16]. Since the first PHD-finger protein HAT3.1 was identified in *Arabidopsis* [17], more and more PHDs have been reported in plants. Until now, the PHD family proteins have been identified in several plant species. A previous study in 2004 identified 45 PHDs in *Arabidopsis* and 44 in rice [18]. Recent research also suggests a total of 73 poplar PHDs [19] and 67 maize PHDs [20]. Current research shows that the plant PHD family was not evolutionarily conserved, but displayed a great diversity in protein sequence, structure and evolutionary relationship [18,19,20].

Consistently, the PHD-finger family also exhibits diverse roles during plant growth and development. For instance, *Arabidopsis* PHD-domain ALFIN1-like proteins are found to promote seed germination [21]. In *Arabidopsis*, PHDs also participates in regulating flowering by modifying the SOC1/FT chromatin conformation [22]. Besides, a barley PHD protein MS1 is suggested to play a central role in pollen development [23]. However, the biological roles of PHDs in stress responses are rarely reported. For now, several studies uncover the differential expression of PHDs under environmental stress. In soybean, the expression of six *GmPHDs* is induced by drought stress. Among them, *GmPHD4*/*5* expression is also up-regulated by cold stress [24]. Moreover, *GmPHD2*/*5* are identified to regulate salt stress responses [24,25]. In maize, 15 of 67 *ZmPHDs* respond to environmental stresses, including drought and salt [20]. Besides, in poplar, nine *PtPHDs* show differential expression under salt, drought and cold stresses [19]. However, no genetic evidence is provided regarding the rice PHD genes in stress responses.

Hence, in this research, we carried out comprehensive analyses of the rice PHD family, identified the phylogenetic relationship, gene duplication, gene structure and functional domains, and further investigated their expression patterns in different tissues and under diverse environmental stresses. Our results will benefit further research concerning the potential roles of the rice PHDs in stress responses.

## 2. Results

### 2.1. Identification and Chromosomal Location of the Rice PHD Family Genes

The PHD-finger proteins play crucial roles in plant growth and development [26]. Hence, in this research, we attempt to identify the rice PHD family genes and investigate their expression profiles. However, due to the extremely high homology of the protein sequences and structures of the PHD, RING and LIM domains, the HMMER search cannot distinguish the PHD, RING and LIM proteins. Therefore, in this research, we identified the PHD genes from the rice genome by combining the results from four searching methods: a “PHD-finger” keyword search and BLASTP search against the Phytozome database, a “PF00628” keyword search against the Rice Genome Annotation Database and an HMMER search against the rice proteome database. The putative candidates were subjected to SMART and Pfam analyses to ensure the existence of the complete PHD domains.

**Figure 1:**
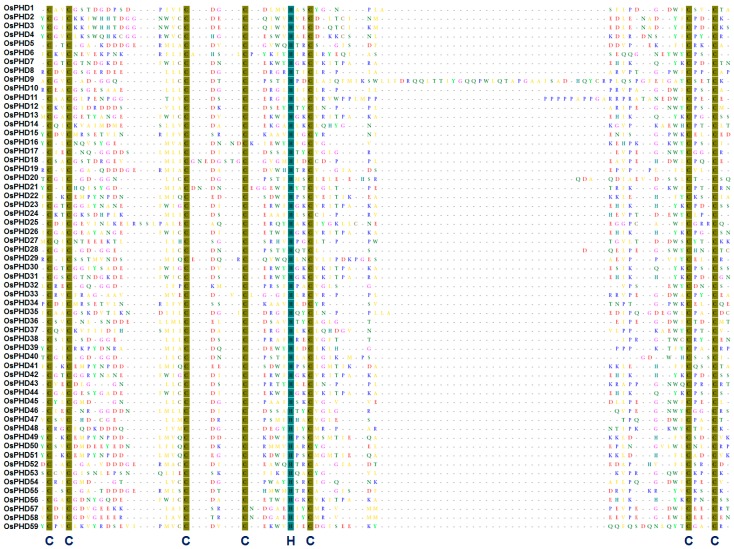
Protein sequence multiple alignment of the PHD-finger domains in the rice PHD family proteins. The multiple alignment was conducted with the amino acid sequences within the predicted PHD domains by using MEGA5.0 software with default parameters. The conserved amino acids (Cys4-His-Cys3) within the PHD-finger domains are shaded in brown and blue.

As a consequence, a total of 59 PHD genes (named as *OsPHD1* to *OsPHD59*) were identified (Table 1) and the C4HC3 sequence motifs within the PHD domains are shown in Figure 1. These PHD family members varied markedly in protein sequence length from 175 (*OsPHD6*) to 2192 (*OsPHD43*) amino acids (aa), with an average length of 722 aa. The molecular masses varied from 19.75 (*OsPHD6*) to 243.66 (*OsPHD43*) kDa and the predicted isoelectric points varied from 4.46 (*OsPHD46*) to 9.87 (*OsPHD38*).

**Figure 2:**
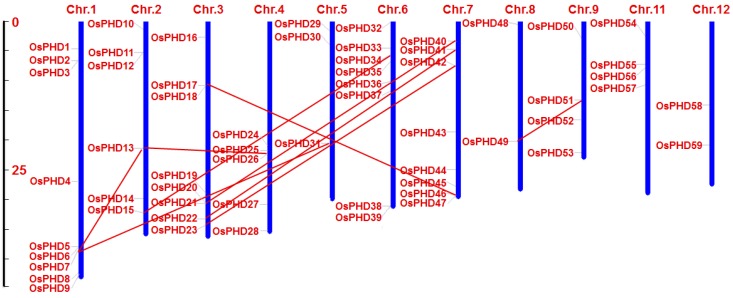
Chromosomal distribution of the rice PHD family genes. The blue bars represent the chromosomes and the chromosome numbers are shown at the top of the bars. The duplicated gene pairs are identified and connected by solid lines. The scale bar on the left represents the length of the chromosome.

Considering the great differences in aa numbers and pI values of OsPHDs, we speculated that the rice PHD family was not as evolutionarily conserved as other protein families. In this context, we performed chromosomal location and synteny analysis of *OsPHDs*. As shown in Figure 2, 59 *OsPHDs* are located on 11 chromosomes except chromosome 10 and they are not randomly or equally distributed on each chromosome. Chromosome 1 possessed nine OsPHDs, while chromosomes 8 and 12 only contained two. Furthermore, synteny analysis revealed only 9 pairs of duplicated genes (Figure 2 and Figure S1), indicating the high divergence of the rice PHD family. Consistently, previous studies reported only 6 duplicated pairs within 73 poplar PHDs [19] and 12 pairs within 67 maize PHDs [20].

### 2.2. Phylogenetic Analyses and Gene Architecture of the Rice PHD Family

**Figure 3:**
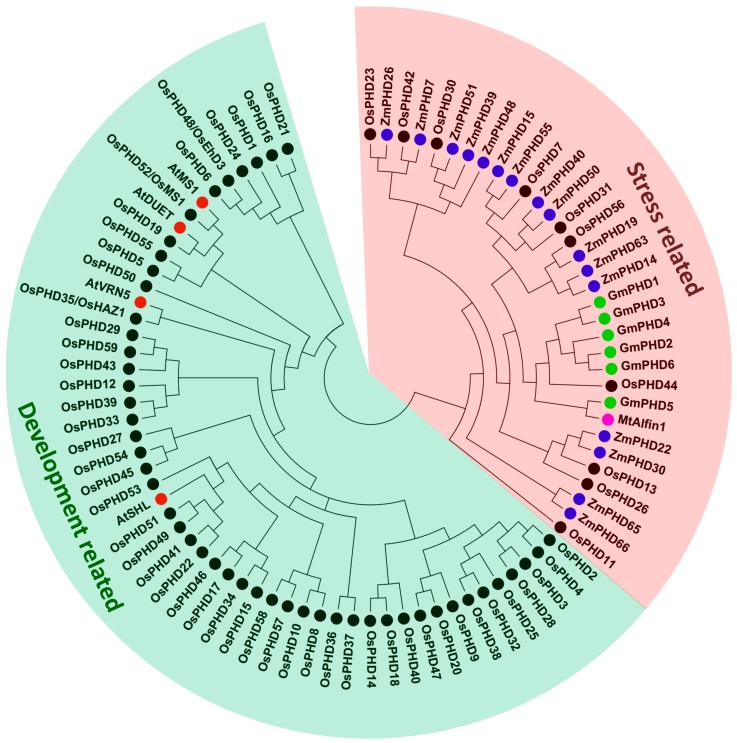
Phylogenetic tree analyses of all OsPHDs proteins and PHDs from other species. The phylogenetic tree was constructed based on the full-length amino acid sequences by using MEGA5.0 software with the Neighbor–Joining method. The red shade marks the stress related cluster that includes 10 *OsPHDs* as well as 6 *GmPHDs* and 16 *ZmPHDs* which are characterized to participate in stress responses. The green shade marks other *OsPHDs* and some PHD proteins from Arabidopsis and alfalfa that are linked to plant growth and development regulation.

To better understand the rice PHD family, we generated a phylogenetic tree by using the full-length protein sequences of OsPHDs as well as some PHDs from other species that have been functionally reported previously. As shown in Figure 3, these PHD proteins are grouped into two major clusters. Out of the 59 *OsPHDs*, ten were clustered together with the stress-responsive PHDs from maize [20], soybean [24,25] and alfalfa [30], indicating the involvement of these *OsPHDs* in stress responses. Another cluster included the other 49 *OsPHDs* and PHDs which were reported to participate in plant reproductive growth and development, such as *AtMS1* [31,32,33], *AtDUET* [34], *AtVRN5* [35], *AtSHL* [36], *OsPHD48*/*OsEhd3* [28], *OsPHD35*/*OsHAZ1* [27] and *OsPHD52*/*OsMS1* [29].

**Figure 4:**
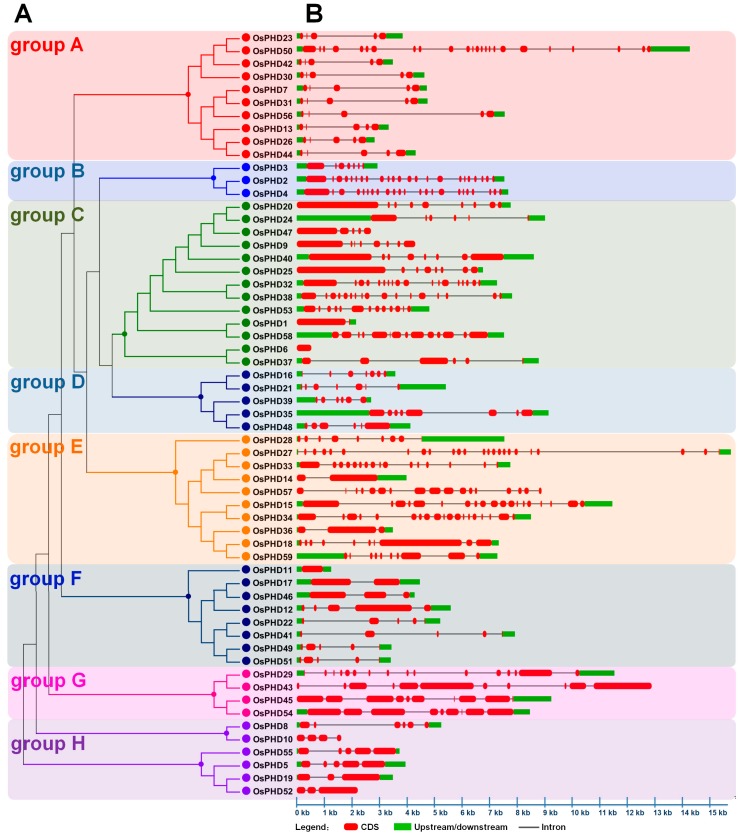
Phylogenetic tree and gene structures of the rice PHD family. (**A**) Phylogenetic tree analyses of the rice PHD family. The phylogenetic tree was constructed based on the full-length amino acid sequences of the rice PHD proteins by using MEGA5.0 software with the Neighbor–Joining method and the bootstrap values were set at 1000. Eight groups (A–H) are marked with different colors; (**B**) Gene structures of the rice PHD family. The gene structures were analyzed by using the Gene Structure Display Server (GSDS2.0). Exons, introns and untranslated regions are indicated by red rounded rectangles, black lines and green boxes, respectively. The scale bar at the bottom is used to estimate the sizes of exons, introns and untranslated regions.

In order to further investigate the evolutionary relationship, the full-length aa sequences of OsPHDs were used to construct an un-rooted phylogenetic tree. As shown in Figure 4A, the rice PHD family could be divided into eight groups (groups A to H). Three of them (groups A/C/E) have more members than the others, with 11 *OsPHDs* in group A, 13 in group C and 10 in group E. However, group B only possessed three PHD proteins. It is worth noting that the duplicated genes described above (Figure 2) were mainly distributed in group A and F (Figure 4A). This finding indicated that members in group A and F were relatively evolutionarily conserved, even though the PHD family displayed a great diversity.

To obtain deeper insights into the structural diversity of *OsPHDs*, we analyzed the characteristics of exon-intron organization. As shown in Figure 4B, the number of exons varied greatly, from 1 (*OsPHD11*) to 24 (*OsPHD27*/*50*), and the exon-intron organization of different groups also differed wildly. The differences in exon numbers and exon-intron organization further suggested the evolutionary diversity of the PHD family. Besides, we also noticed that members within each group exhibited a certain similarity in terms of genomic structure. For example, most members from group A or F possessed five exons. Except *OsPHD50*, members in group A showed similar exon-intron composition form. These results further confirmed the finding that although the rice PHD family diverged greatly among different groups, members within each group were relatively conserved.

### 2.3. Investigation of the Conserved Functional Domains in OsPHD Proteins

**Figure 5:**
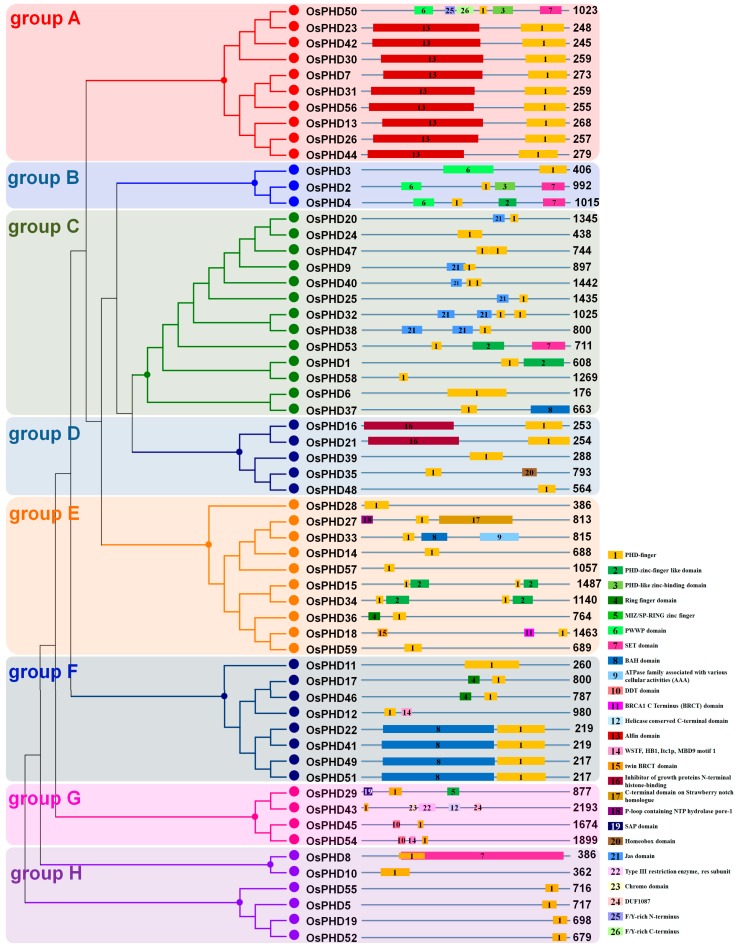
Functional domains of the rice PHD family proteins. The information on functional domains was obtained from the phytozome and is shown in the diagrams. Different colored boxes with numbers in them represent different functional domains.

To further verify the diversity of the rice PHD family, we analyzed the functional domains of all the rice PHD proteins (Figure 5). Our results showed that members within each group shared high similarity in domain structure, while members from different groups varied widely, indicating the potential functional divergence and specialization of *OsPHDs* from different groups.

In detail, with the exception of *OsPHD50*, members in group A contained an Alfin domain (PF12165). Alfin-containing PHD proteins acted as a histone-binding component which specifically recognized H3K4me3 and were reported to participate in salt stress responses [30,37]. This finding indicated the involvement of group A *OsPHDs* in salt stress responses. Four members in group F, as well as *OsPHD33* in group E and *OsPHD37* in group C, possessed a BAH domain (PF01426). The BAH domain functioned through protein-protein interaction and was linked to DNA methylation and gene silencing [38,39]. Furthermore, a SET domain (PF00856) was found in five *OsPHDs*. The SET domain was reported to regulate gene transcription and chromatin structure, and participate in growth control [40,41]. In addition, members in group B and *OsPHD50* in group A also contained a PWWP domain (PF00855), which is involved in protein-protein interaction with other histone and DNA modifier domains [42,43]. These results implied that *OsPHDs* might function in epigenetic regulation of gene expression. Moreover, six members in group C contained a Jas domain (PF16135), which plays a crucial role in the jasmonate signalling pathway. In conclusion, the variety of functional domains suggested the putative functional diversity of *OsPHDs* from different groups.

To further confirm the functional divergence of *OsPHDs*, we then investigated the conserved motifs by using MEME with the number of motifs set to be 20 (Figure S2). Among the 20 motifs, motifs 2, 4, 14 and/or 20 representing the PHD-finger domain were present in all groups. Motifs 1 and 7, constituting the Alfin domain, were specific to group A. Motifs 19, 16 and 10 were shared by members in group B, while motifs 3, 17 and 18 were present in *OsPHDs* from group C. Motifs 8 and 5 were observed in *OsPHDs* from group F, while motifs 15 and 6 were specific to group H. Motif 9 was only presented in *OsPHD57* in group E and *OsPHD58* in group C. Motifs 11 and 12 existed in *OsPHD50* in group A and *OsPHD15*/*34* in group E, while motif 13 was only presented in *OsPHD17* and *OsPHD46* in group F. These results indicated that *OsPHDs* in the same group shared similar motifs, while motifs are divergently distributed among specific groups, which might contribute to the functional divergence of the rice PHD family.

### 2.4. Expression Profiles of OsPHD Genes in Rice Tissues

**Figure 6:**
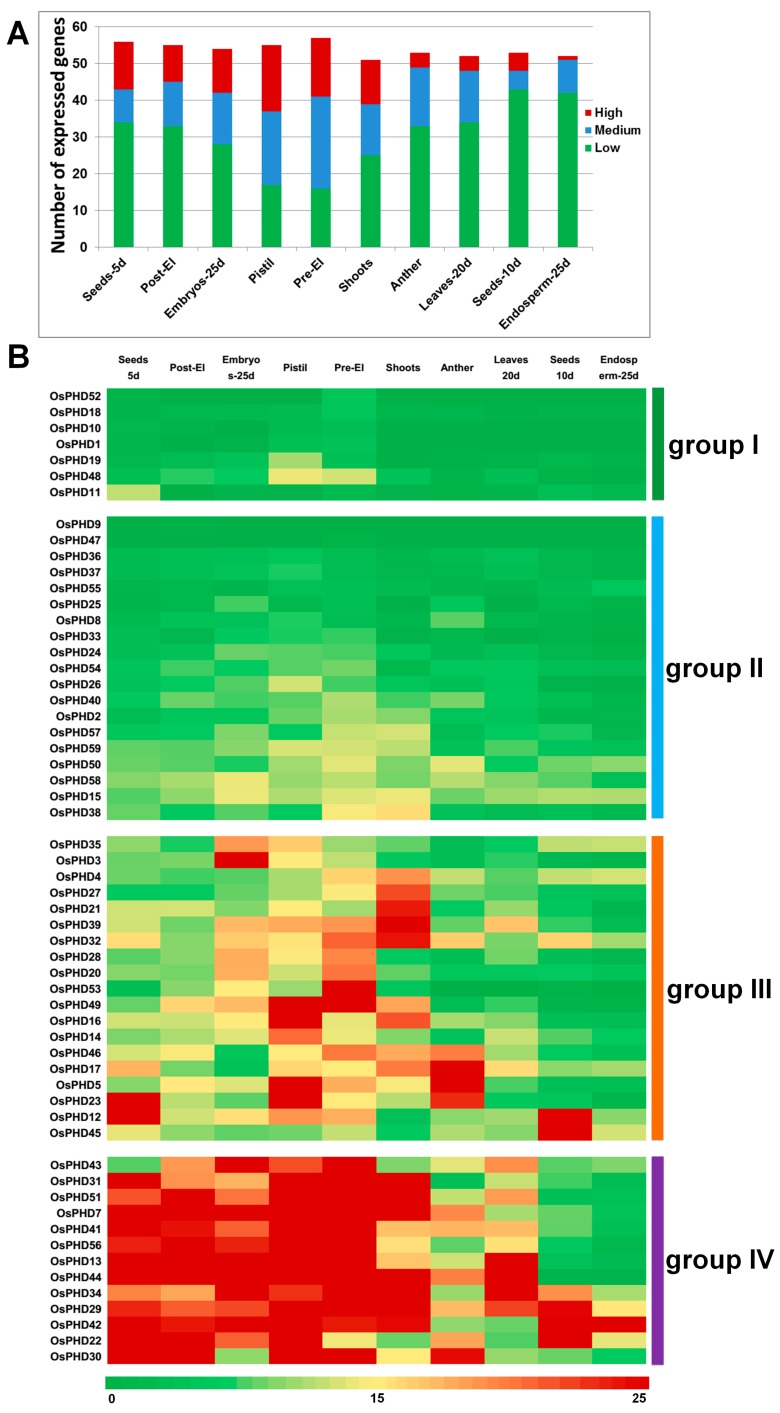
Expression of the rice PHD family genes in different tissues. (**A**) Numbers of expressed genes in different tissues. Expression data in different tissues were downloaded from the Rice Expression Profile Database. High: expression values >20, medium: 20 ≥ expression values > 10, low: 10 ≥ expression values > 0; (**B**) Expression profiles of the rice PHD family genes in different tissues. A heat map was generated and *OsPHDs* were manually clustered into four groups according to their expression values in different tissues. The color scale represents the expression values: red indicates high levels and green represents low levels. Pre-EI: pre-emergence inflorescence; Post-EI: post-emergence inflorescence.

In an attempt to explore the potential function of *OsPHD* family genes, we retrieved the expression profiles from RiceXPro and analyzed their expression in different tissues. As shown in Figure 6A, the numbers of expressed *OsPHDs* in different tissues varied little, from 51 to 57. However, the numbers of *OsPHDs* with high, medium or low expression levels varied greatly. Notably, *OsPHDs* expressed at relatively higher levels in pre-emergence inflorescence and pistils, with less lowly-expressed genes. In contrast, the numbers of lowly-expressed *OsPHDs* were obviously greater in seeds-10d (d: day. The same below.) and endosperm-25d. Furthermore, the numbers of highly-expressed *OsPHDs* were smaller in endosperm-25d (1), anther (4) and leaves-20d (4).

According to their expression characteristics in different tissues, *OsPHDs* were clustered into four groups (groups I to IV, Figure 6B). *OsPHDs* in groups I and II expressed at lower levels in all tissues, but members in group I displayed relatively higher expression in a specific tissue. For instance, although *OsPHD52*/*OsMS1* showed very low expression levels in most tissues, its expression displayed a dominant enrichment in pre-emergence inflorescence (Figure 6B, Figure S3A). Furthermore, the specific expression of *OsPHD52*/*OsMS1* in pre-emergence inflorescence was in line with its regulatory role in pollen development [29]. Moreover, *OsPHD48*/*OsEhd3* showed relatively higher expression in pistils and pre-emergence inflorescence, which is consistent with previous reports that *OsEhd3* is a critical promoter of rice flowering [28].

Group III members displayed typical tissue specific expression, but expressed at higher expression values than group I. For example, *OsPHD12*/*45* were highly expressed in seeds-10d, while *OsPHD3* was highly expressed in embryos-25d. Notably, with the exception of *OsPHD23*, all group III *OsPHDs*, showing tissue specific expression patterns, belonged to the development related cluster as described in Figure 3. Remarkably, *OsPHD35*/*OsHAZ1* from group III, which participates in radial axis differentiation in a globular embryo [27], showed the highest expression in embryos-25d. Interestingly, members in group III did not show high expression in either post-emergence inflorescence, leaves-20d or endosperm-25d. In contrast, *OsPHDs*, which highly expressed in these three tissues, were all clustered into group IV. Besides, most members in group IV were highly expressed in more than three tissues. In conclusion, these results suggested that *OsPHDs* displayed tissue-specific expression profiles, indicating their potential divergent roles in plant development.

### 2.5. Expression Responses of OsPHD Genes to Environmental Stresses

**Figure 7:**
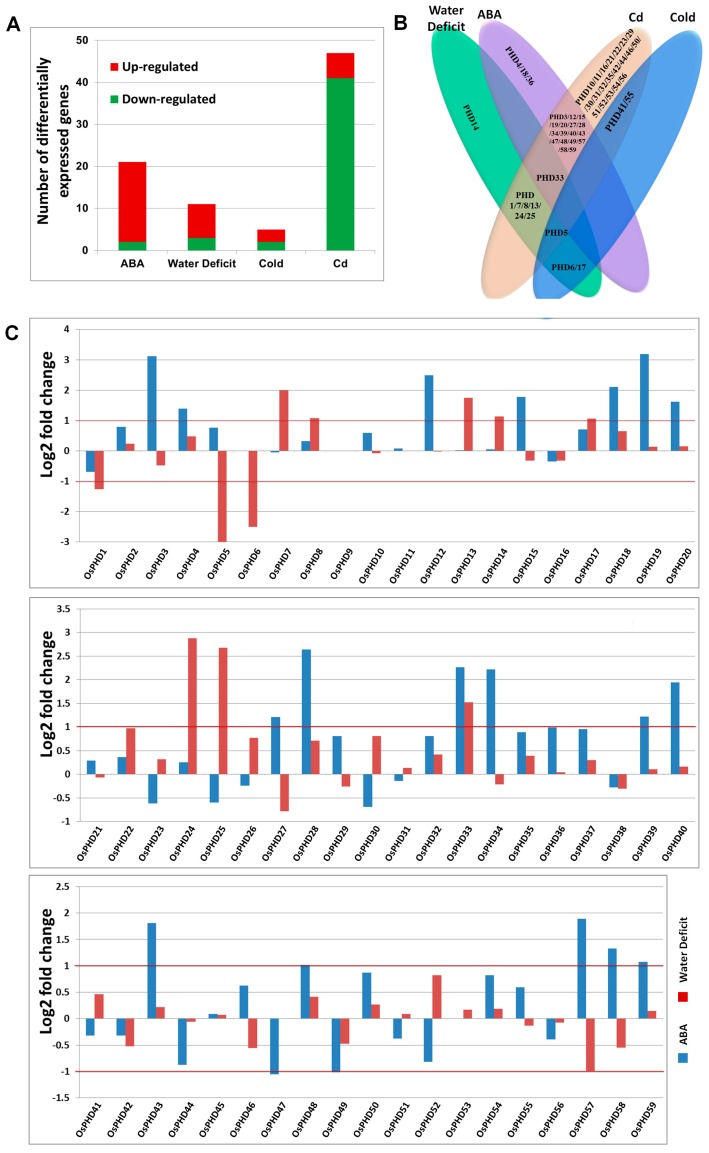
Expression analyses of the rice PHD family genes under different environmental stresses. (**A**) Numbers of differentially expressed *OsPHD* genes in stress responses. Expression data in different tissues were downloaded from the Rice Expression Profile Database. Compared with the control, genes with |Log2 fold change| > 1 were designated as differentially expressed genes; (**B**) Venn diagram depicting the overlap of differentially expressed genes under different environmental stresses; (**C**) Expression of *OsPHDs* under ABA and water deficit stresses. The Y-axis represents the log2 of fold changes. The red lines mark the cut-off lines (|Log2 fold change| = 1).

In order to investigate the potential roles of *OsPHDs* in environmental stress responses, we further analyzed their expression under ABA (Accession No. SRX332134 and SRX330497), high Cd (DRX012208-DRX012225) and water deficit (SRX844621-SRX844624) by using the transcript data from RiceXPro, as well as our previous microarray data under cold stress (Table S1). As shown in Figure 7A, 47 of the 59 *OsPHD* genes are differentially expressed (|Log2 fold change| > 1) under high Cd stress, while only 5 *OsPHDs* display altered expression under cold stress. Furthermore, the expression of 21 and 11 *OsPHDs* was differentially regulated by ABA and water deficit, respectively. Interestingly, most *OsPHDs* were down-regulated under high Cd concentration, while under ABA and water deficit, the numbers of up-regulated *OsPHDs* were obviously larger than that of down-regulated. Notably, 29 of these *OsPHD* genes showed differential expression under two or three types of stresses (Figure 7B). Among them, *OsPHD33* expression responded to ABA, water deficit and Cd stresses, and *OsPHD5* was differentially expressed under ABA, water deficit and Cd stresses. However, no *OsPHD* gene was observed to be differentially expressed in response to all four types of stresses.

In detail, after ABA treatment, 19 *OsPHD* genes were up-regulated, while only two were down-regulated (Figure 7C). Among the up-regulated *OsPHD* genes, *OsPHD3* and *OsPHD19* displayed the greatest ABA-induced expression, with Log2 fold change >3. It is worth noting that most members in group B and E showed differential expression under ABA stress, indicating that they may be involved in ABA signaling transduction and/or stress responses. Compared with ABA stress, less *OsPHD* genes with differential expression were observed under water deficit (Figure 7C). Only eight up-regulated and three down-regulated *OsPHDs* were found when the soil water content decreased to 40% of that under normal conditions. Among them, *OsPHD5* showed the greatest decreased expression, while *OsPHD24* exhibited the largest increased expression. Interestingly, by comparing the differentially expressed *OsPHDs* under ABA and water deficit stress, we found that only *OsPHD33* showed differential expression under both stresses, while other *OsPHDs* only differentially expressed under one type of stress. This finding implied that *OsPHD* genes might participate in water deficit stress responses through ABA-independent ways.

**Figure 8:**
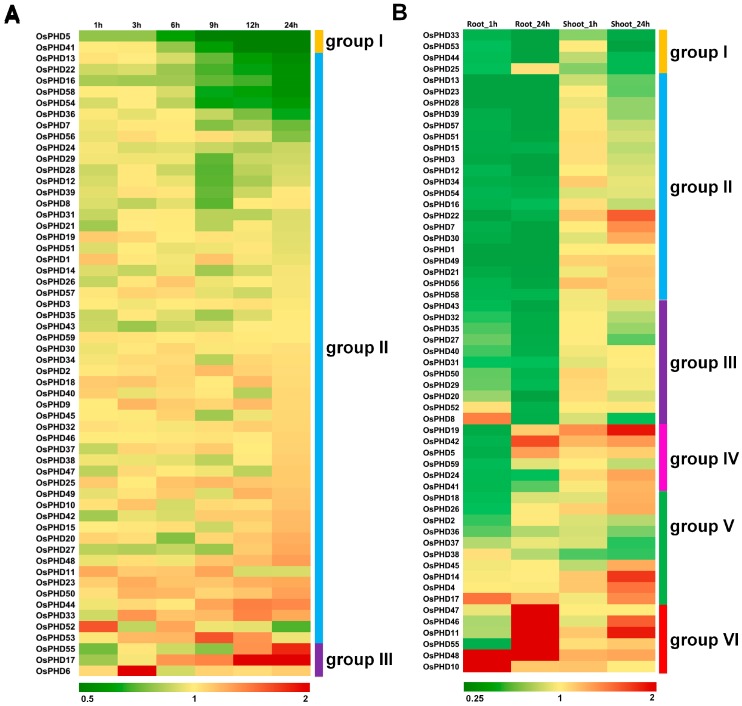
Expression profiles of the rice PHD family genes under cold (**A**) and Cd (**B**) stresses. Expression data were downloaded from the Rice Expression Profile Database. A heat map was generated and *OsPHDs* were manually clustered into different groups according to their expression values. The color scale represents the relative expression values compared with the control. Red indicates high levels and green represents low levels.

In previous research, we carried out the microarray analysis by using rice seedlings which were exposed to 4 °C for 1, 3, 6, 9, 12 and 24 h. Based on the expression profiles under cold stress, the rice *PHD* family was divided into three groups (groups I–III, Figure 8A). Group I only contained two *OsPHDs* showing decreased expression after 4 °C treatment, while group III included three *OsPHDs* with increased expression. Most *OsPHDs* genes were clustered into group II and their expression was not influenced by cold stress. Notably, with the exception of *OsPHD6*, all these differentially expressed members belonged to group F and H, implying the potential involvement of group F and H *OsPHDs* in cold stress responses.

In contrast, 47 out of 59 *OsPHDs* were differentially expressed when rice roots were immersed in 50 µM Cd solution (for 1 and 24 h) and most of them were down-regulated (Figure 8B). What is interesting is that *OsPHDs* showed extremely distinctive expression patterns between roots and shoots. We checked the raw data of *OsPHDs* expression at 0, 1 and 24 h under Cd stress (Figure S4). By comparing the data in roots and shoots at 0 h, we found that under normal conditions, most *OsPHDs* displayed similar expression levels in roots and shoots (Figure S4B). After Cd treatment, most *OsPHDs* expression changed in the roots, while few *OsPHDs* showed altered expression in the shoots. This finding suggested that the difference of *OsPHDs* expression patterns between roots and shoots is cadmium related. In detail, after 50 µM Cd treatment, 47 *OsPHDs* were differentially expressed in the roots, while only four were differentially expressed in the shoots. According to their expression patterns, *OsPHDs* were divided into six groups (groups I–VI, Figure 8B). The genes in group I showed decreased expression in both roots and shoots, while expression of *OsPHDs* in groups II–IV was inhibited only in the roots. Group II members were down-regulated at both 1 and 24 h, while expression of groups III and IV *OsPHDs* was decreased only at 24 and 1 h, respectively. Furthermore, expression of members in group V was not affected, however group VI *OsPHDs* were up-regulated under Cd stress in the roots. In conclusion, the differential expression of *OsPHDs* under diverse environmental stresses suggested their potential roles in plant stress responses.

### 2.6. Quantitative Real-Time PCR Analyses of OsPHDs Expression in Response to Environmental Stresses

**Figure 9:**
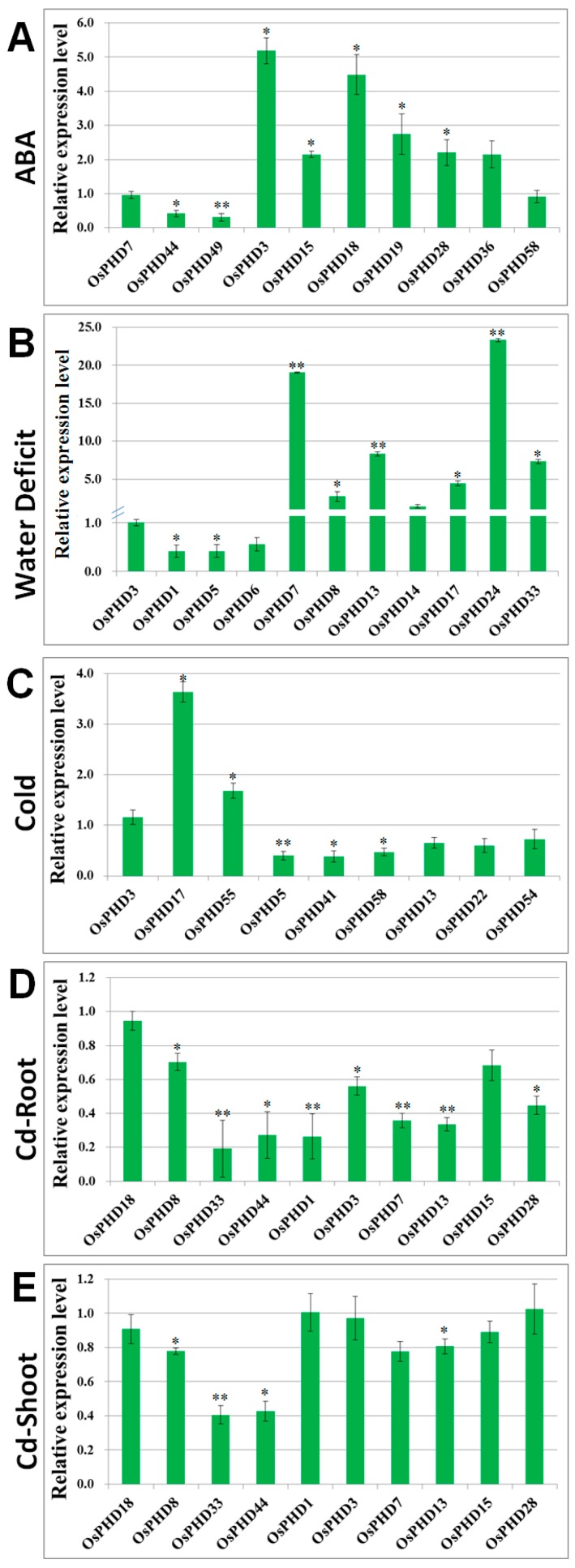
Quantitative real-time PCR validation of *OsPHDs* under ABA (**A**); water deficit (**B**); cold (**C**); Cd-root (**D**) and Cd-shoot (**E**) stresses. For ABA and Cd treatments, the three-week-old rice seedlings were treated with 50 µM ABA or 50 µM Cd for 24 h. For cold stress, rice seedlings were exposed to 4 °C for 24 h. For water deficit stress, water irrigation was stopped until the soil water content was decreased to 40% of that under normal conditions. For each stress treatment, one *OsPHD* gene whose expression was not affected by this stress, was used as a reference for qRT-PCR assays. *OsEf1-α* (elongation factor 1-α) was used as an internal reference. The mean values (±SE) from three fully independent biological repeats and three technical repeats are shown. Asterisks indicate significant differences (* *p* < 0.05; ** *p* < 0.01 by Student’s *t*-test).

In order to further verify the stress induced expression of *OsPHDs*, we focused on several representative genes showing differential expression under stress treatment and conducted quantitative real-time PCR analyses. For each stress treatment, we selected one PHD gene whose expression was not affected by this stress, as a reference for qRT-PCR assays. As for ABA stress, expression of two down-regulated and seven up-regulated *OsPHDs* is verified (Figure 9A). Specifically speaking, *OsPHD44* and *OsPHD49* displayed decreased expression, and five *OsPHDs* exhibited increased transcript levels under ABA stress. Under water deficit stress, expression of two *OsPHDs* (*OsPHD1* and *OsPHD5*) was reduced, while transcript levels of six other *OsPHDs* were induced (Figure 9B).

Besides, we also confirmed the differential expression of *OsPHDs* after cold treatment for 24 h (Figure 9C). Expression of two down-regulated (*OsPHD5* and *OsPHD41* in group I) and two up-regulated (*OsPHD55* and *OsPHD17* in group III) was verified by quantitative real-time PCR results. We also investigated the transcript levels of four members belonging to group II, which showed slightly decreased expression at 24 h. Results illustrated that only *OsPHD58* expression showed a slight decrease with statistical significance after cold stress. Expression of the other three *OsPHDs* was slightly reduced, but with no statistical significance. Moreover, as for Cd stress, we also selected nine down-regulated *OsPHDs* from groups I and II, and validated their decreased expression under Cd stress in roots (Figure 9D). With the exception of *OsPHD15*, all of them were down-regulated by Cd stress in roots, however, only four of them displayed decreased expression in shoots.

In conclusion, our qRT-PCR results confirmed that *OsPHD1*/*7*/*8*/*13*/*33* were differentially expressed under water deficit and Cd stress, while *OsPHD5*/*17* showed altered expression under water deficit and cold stress. Moreover, *OsPHD3*/*44*/*28* expression was differentially regulated by both ABA and Cd stresses. To conclude, quantitative real-time PCR results presented here suggest that the rice PHD family genes possibly participate in plant responses to diverse environmental stresses.

## 3. Discussion

Previous studies have demonstrated that PHD proteins play crucial roles in plant growth and development [21,22,23,26]. However, the biological roles of PHDs in regulating plant stress tolerance are rarely studied. Hence, in this research, we conducted a comprehensive investigation of the rice PHD family, trying to identify potential *OsPHDs* that responded to environmental stresses.

The PHD-finger family was reported to be evolutionarily divergent [18,19,20]. Here, in this research, we further provided several pieces of evidence to show the divergent evolution of the rice PHD family. Firstly, the phylogenetic tree showed that the rice PHD family could be divided into eight groups (Figure 4A). Previous studies reported that the poplar and maize PHDs could be clustered into 11 and 10 subfamilies, respectively [19,20]. Notably, several PHDs could not be clustered into any subfamily due to the low bootstrap values. Secondly, the exon numbers of the rice PHDs varied greatly, from 1 (*OsPHD11*) to 24 (*OsPHD27*/*50*), and the gene structure also differed wildly among different PHD groups (Figure 4B), which further suggested the evolutionary diversity of the rice PHD family. Similarly, maize PHDs from different subfamilies also showed a great diversity in terms of intron numbers and exon length [20]. Thirdly, the rice PHDs varied markedly in protein sequence length, from 175 (*OsPHD6*) to 2192 aa (*OsPHD43*). Investigation of functional domains and conserved motifs further revealed that members from different PHD groups also varied widely in domain architecture (Figure 5) and some motifs were exclusively found in a particular group (Figure S1). This finding is in line with previous reports [19,20] and further suggests the diversity of the plant PHD family. Lastly, synteny analyses only identified nine pairs of duplicated PHD genes (Figure 2). Consistently, only six duplicated pairs were observed within 73 poplar PHDs [19] and 12 pairs within 67 maize PHDs [20]. All of the above results support the evolutionary divergence of the rice PHD family.

Even though the rice PHD family is evolutionarily divergent, some PHD groups are relatively conserved. For instance, investigation of gene structure (Figure 4B), functional domains (Figure 5) and conversed motifs (Figure S1) illustrated that *OsPHDs* within group A and F exhibited high similarity. Consistently, the duplicated *OsPHDs* genes (Figure 2) were mainly distributed in group A and F (Figure 4A). Similar findings were also observed for Subfamily IX PHDs in maize [20]. The conservation of PHD proteins within certain subfamilies indicated similar and/or overlapping function of these PHD genes. For example, in maize, expression of Subfamily IX *ZmPHDs* responded to salt, drought and ABA stresses [20]. In addition, phylogenetic analysis of *OsPHDs* with PHDs from other species (Figure 3) showed that group A *OsPHDs* were grouped into the same cluster together with Subfamily IX *ZmPHDs* and six *GmPHDs*, which were reported to respond to environmental stress [20,24,25]. This finding indicates that although the PHD family is divergent in plants, some PHD subfamilies are relatively conserved among different species.

On the other hand, the structural divergence of different OsPHD groups might contribute to the functional specialization of the rice PHD family. Among the 59 *OsPHDs*, 26 genes (groups I and III in Figure 6B) tended to specifically express in certain tissues. Notably, with the exception of *OsPHD23*, all *OsPHDs* showing tissue specific expression patterns belonged to the development related cluster as described in Figure 3. Among them, *OsPHD52*/*OsMS1* is closely related to *AtMS1* in the phylogenetic tree (Figure 3) and its expression displayed a dominant enrichment in pre-emergence inflorescence, which is in line with its regulatory role in pollen development [29]. *OsPHD48*/*OsEhd3*, which is a critical promoter of rice flowering [28], showed relatively higher expression in pistils and pre-emergence inflorescence. Moreover, *OsPHD35*/*OsHAZ1*, which was previously reported to participate in radial axis differentiation in a globular embryo [27], showed the highest expression in embryos-25d. Remarkably, in the phylogenetic tree (Figure 3), *OsPHD35*/*OsHAZ1* is the closest one to *AtVRN5*, which was found to function in the epigenetic silencing of *Arabidopsis FLC* during flowering [35]. In addition, in the phylogenetic tree (Figure 3), *OsPHD19* appeared in a pair with *AtDUET*, which is involved in male meiosis [34]. Consistently, *OsPHD19* displayed high expression in pistils. *OsPHD53*/*49*, showing high expression in pre-emergence inflorescence, were closely related to *AtSHL*, which is required for proper development and fertility [36]. Taken together, these findings strongly suggest the potential role of *OsPHDs* in reproductive growth and development.

In addition to plant growth and development, PHDs were also shown to be involved in stress responses [24,25,44]. Our results also uncovered the differential responses of *OsPHDs* expression to ABA, high Cd, water deficit and cold stresses (Figure 7, Figure 8 and Figure 9). Among the four types of environmental stresses detected in this research, more *OsPHDs* with differential expression were observed under 50 µM Cd treatment. Notably, Cd stress mainly inhibited *OsPHDs* expression, as evidenced by 41 down-regulated but 6 up-regulated genes (Figure 7A and Figure 8B). In contrast, under ABA and water deficit stresses, more *OsPHDs* showed up-regulated expression (Figure 7). This difference indicates that *OsPHDs* might respond to diverse stresses through different ways.

ABA, as a crucial plant hormone, plays a pivotal role in environmental stress responses [45,46,47]. In this research, we showed that ABA stress greatly triggered the expression of *OsPHDs* (Figure 7C). By quantitative real-time PCR analysis, we validated that after 50 µM ABA treatment for 24 h, the transcript levels of seven *OsPHDs* were obviously increased in rice leaves (Figure 9A). Previous research also reported that in maize, 14 PHD genes within Subfamily IX were up-regulated when three-week-old seedling leaves were sprayed with 100 μM ABA solution. The ABA responsive elements (ABREs) were found in promoter regions of these PHD genes [20]. Similarly, when soybean seedling roots were immersed in 100 µM ABA, *GmPHDs* expression was also induced, especially in the stress-tolerant cultivar JD23 [24]. These findings suggested the putative roles of the PHD family genes in ABA signal transduction and responses. However, in our research, even though group A *OsPHDs* were grouped into the same cluster with Subfamily IX *ZmPHDs* and six *GmPHDs* (Figure 3), most of them did not show differential expression under ABA and water deficit stresses. Further experiments are needed to illustrate the difference.

ABA has pivotal roles in plant tolerance to water deficit or drought stress [48,49]. Considering the differential expression of *OsPHDs* under ABA treatment, we also investigated their expression under water deficit stress. Compared with ABA, fewer *OsPHDs* genes with differential expression were observed in three-week-old rice leaves when the soil water content was decreased to 40% of that under normal conditions (Figure 7C). The altered expression of *OsPHDs* was shown by quantitative real-time PCR results (Figure 9B). Among them, *OsPHD24* and *OsPHD25* exhibited dramatically increased expression, while *OsPHD5* and *OsPHD6* showed greatly decreased expression. Similarly, *GmPHDs* expression was also increased in soybean seedlings which were placed on Whatman filter paper for 3 h for dehydration [24]. The responsive expression of PHD genes under drought stress was also observed in maize and poplar [19,20]. After 20% PEG6000 treatment, the PHD genes in maize and poplar were greatly induced. In addition, the dehydration responsive elements (DREs) were found to be enriched in promoters of drought responsive *ZmPHDs* [20]. However, among the differentially expressed *OsPHDs* under ABA and water deficit stresses, most of them only differentially expressed under one type of stress, while only *OsPHD33* showed altered expression under both stresses. This finding implies that *OsPHDs* might participate in water deficit stress responses through ABA-independent ways. Taken together, the above results imply that these PHD genes might take part in water deficit tolerance.

In contrast, when exposed to 4 °C, only five *OsPHDs* showed altered expression (Figure 8A), which was further supported by the quantitative real-time PCR results (Figure 9C). With the exception of *OsPHD6*, all of them belonged to groups F and H, implying the involvement of these two groups in cold stress responses. Among them, *OsPHD6*/*17*/*55* were up-regulated, while *OsPHD5*/*41* were down-regulated, which is in line with previous research. In poplar, after exposure to 4 °C, expression of *PtPHD12*/*68*/*36*/*50* was decreased, but transcript levels of *PtPHD21*/*29*/*31*/*32*/*65* were increased [19].

Compared with cold stress, Cd stress greatly altered the expression levels of 41 *OsPHDs* genes (Figure 8B). Most of them were down-regulated by Cd stress, which was further verified by the quantitative real-time PCR results (Figure 9D–E). This finding indicates that the PHD family genes possibly play important roles in rice responses to Cd stress. Interestingly, in roots, 47 *OsPHDs* were differentially expressed, however, in shoots, only four *OsPHDs* displayed differential expression (Figure 8B). Under normal conditions, most *OsPHDs* genes displayed similar expression levels between roots and shoots (Figure S4). After Cd treatment, most *OsPHDs* expression in roots changed, while few *OsPHDs* showed altered expression in shoots. This finding suggests that the difference of *OsPHDs* expression patterns between roots and shoots is cadmium related. This difference implies different responsive mechanisms between roots and shoots, and it is possible that *OsPHDs* mainly function in roots.

Taken together, current studies have demonstrated the key roles of PHDs in plant growth and development. A handful of studies reported the involvement of PHD in salt stress. However, no biological evidence was given to show PHD function in other environmental stresses. In this research, we suggested that expression of *OsPHDs* was responsive to ABA, water deficit, cold and especially Cd stress. Hence, it will be interesting to study the responsive mechanisms and potential roles of *OsPHDs* in response to these environmental stresses.

## 4. Materials and Methods

### 4.1. Identification of the PHD-Finger Family Genes in Rice

To identify the candidate PHD genes in the rice genome, we downloaded the HMM profile of the PHD proteins from the HMMER website (https://www.ebi.ac.uk/Tools/hmmer/search/hmmsearch) and used it to search against the rice proteome database. We also conducted a BLASTP search against rice proteome. Furthermore, “PHD-finger” as a keyword was submitted to search Phytozome (https://phytozome.jgi.doe.gov/pz/portal.html#) [50] and “PF00628” was used as a keyword to search against the Rice Genome Annotation Database (http://rice.plantbiology.msu.edu/index.shtml). The amino acid sequences of candidates from these four searches were screened by SMART (http://smart.emblheidelberg.de) [51] and Pfam (http://pfam.sanger.ac.uk/) [52] to remove proteins without a complete PHD-finger domain. The information on the rice PHD genes, including locus ID, chromosome locations, ORF length and protein length were obtained from the Phytozome database. The isoelectric point and molecular weight were estimated by using the ExPASy (http://expasy.org/) [53].

### 4.2. Multiple Alignment, Phylogenetic Analysis and Chromosomal Location of the Rice PHD Family Genes

The amino acid sequences within the predicted PHD-finger domains were used for multiple alignment to show the existence of the PHD domains. For phylogenetic analysis, multiple alignment was performed with the full-length amino acid sequences of the rice PHD family proteins by using MEGA5 software [54]. The phylogenetic tree was constructed by using the Neighbor–Joining method with the following parameters: poisson correction, pair-wise deletion and 1000 bootstrap replicates. The chromosomal location of the rice PHD family genes was depicted using the MapInspect software. The synteny blocks of the rice genome were downloaded from the Plant Genome Duplication Database (PGDD, http://chibba.agtec.uga.edu/duplication/) [55] and the duplicated OsPHD pairs were connected by solid lines.

### 4.3. Analyses of Gene Structure, Functional Domains and Conserved Motifs of the Rice PHD Family

The exon and intron structures of the rice PHD family genes were illustrated by using the Gene Structure Display Server (GSDS, http://gsds.cbi.pku.edu.cn/index.php) [56]. The information on functional domains was derived from Phytozome (https://phytozome.jgi.doe.gov/) and SMART. The conserved motifs in the rice PHD proteins were predicted by using the on-line MEME procedure (Multiple Em for Motif Elicitation, http://meme-suite.org/tools/meme) by using the following parameters: 5 ≤ optimum motif width ≤ 200; the number of motifs =20; zero or one occurrence per sequence [57].

### 4.4. Expression Analysis of the Rice PHD Family Genes in Diverse Tissues and Under Different Stress Treatments

The expression data of the rice PHD family genes were downloaded from the Rice Expression Profile Database (RiceXPro, http://ricexpro.dna.affrc.go.jp/) [58]. The transcript data were derived from ten tissues, including leaves-20d (Accession number: SRX100741), shoots (SRX020118), pre-emergence inflorescence (SRX100743), post-emergence inflorescence (SRX100745), pistils (SRX100747), anthers (SRX100746), seeds-5d (SRX100749), seeds-10d (SRX100755), embryos-25d (SRX100753) and endosperm-25d (SRX100754). The accession numbers of transcript data were SRX844621-SRX844624 for water deficit stress, DRX012208-DRX012225 for Cd stress and SRX332134 and SRX330497 for ABA stress.

### 4.5. Quantitative Real-Time PCR Analyses of the Rice PHD Family Genes in Response to Environmental Stresses

Rice seeds were surface sterilized in 10% NaClO, washed with sterile water 3–5 times and placed on moist filter paper for 2–3 days to promote germination. The young seedlings were transformed and grown in Yoshida’s culture solution or in pots filled with a mixture of peat moss: soil (1:1) under a 12 h light (28 °C)/12 h dark (22 °C) photoperiod.

For ABA and Cd treatments, the roots of the three-week-old hydroponic rice seedlings were immersed in Yoshida’s culture solution containing either 50 µM ABA [59,60,61] or 50 µM Cd for 24 h [62]. For cold stress, rice seedlings were exposed to 4 °C for 24 h. For water deficit stress, water irrigation was stopped (for 7 days) until the soil water content was decreased to 40% of that under normal conditions. 

Equal amounts of leaves (for ABA, cold, water deficit and Cd stresses) or roots (for Cd stress) were harvested. Total RNA was extracted using TRIzol^®^ Reagent (Invitrogen, Carlsbad, CA, USA) and cDNA was synthesized by using the SuperScript™ III Reverse Transcriptase kit (Invitrogen, Carlsbad, CA, USA). Quantitative real-time PCR was performed using the CFX96 Touch™ Real-Time PCR Detection System and TransStart Top Green qPCR SuperMix (Beijing TransGen Biotech, Beijing, China). *OsEf1-α* (elongation factor 1-α) was used as an internal reference. Expression levels were calculated and normalized as described [63]. Three independent biological replicates and three technical repeats were carried out. Gene specific primers for quantitative real-time PCR are listed in Table S2.

## 5. Conclusions

In conclusion, we identified a total of 59 PHD family genes in the rice genome. We investigated their chromosomal location, gene duplication, phylogenetic classification, gene structure, functional domains and conserved motifs. Through the comprehensive analyses, we showed that the rice PHD family was evolutionarily divergent among different groups. Meanwhile, we found that some *OsPHDs* were specifically expressed in certain tissues, indicating the involvement of *OsPHDs* in developmental regulation. Moreover, we also uncovered the differential responses of *OsPHDs* expression to environmental stresses, including ABA, water deficit, cold and Cd stresses. By using quantitative real-time PCR, we further confirmed the differential expression of *OsPHDs* under these environmental stresses. Results presented here will be of great importance to further characterize their biological roles in response to environmental stresses.

## Figures and Tables

**Table 1:** Detailed information of all PHD family genes identified in the rice genome.

Name	TIGR Locus ID	Chr.	Location	Open Reading Frame Length (bp)	Protein			Alias
					Amino Acids (aa)	Molecular Weight (KD)	Isoelectric Point	
*OsPHD1*	LOC_Os01g08820	1	4431532–4433679 (R)	1824	607	63.23	8.01	*OsPhd3* [18]
*OsPHD2*	LOC_Os01g11952	1	6507356–6514891 (R)	2976	991	110.89	8.18	
*OsPHD3*	LOC_Os01g11960	1	6518127–6521046 (R)	1218	405	44.46	5.30	
*OsPHD4*	LOC_Os01g46700	1	26591955–26599634 (F)	3048	1015	115.46	6.40	
*OsPHD5*	LOC_Os01g65600	1	38081608–38085548 (F)	2151	716	79.23	7.44	*OsPhd4* [18]
*OsPHD6*	LOC_Os01g66070	1	38344410–38344937 (F)	528	175	19.75	4.84	*OsPhd5* [18]
*OsPHD7*	LOC_Os01g66420	1	38566147–38570861(R)	819	272	29.57	5.32	*OsPhd7* [18]
*OsPHD8*	LOC_Os01g73460	1	42567843–42573082 (F)	1158	385	43.48	8.92	*OsPhd2* [18]
*OsPHD9*	LOC_Os01g73480	1	42579342–42583645 (F)	2694	897	95.02	9.65	
*OsPHD10*	LOC_Os02g03030	2	1190846–1192461 (F)	1086	361	39.59	9.16	*OsPhd11* [18]
*OsPHD11*	LOC_Os02g09910	2	5131380–5132629 (F)	780	259	26.93	5.78	
*OsPHD12*	LOC_Os02g09920	2	5145548–5151131 (F)	2940	979	110.22	4.85	
*OsPHD13*	LOC_Os02g35600	2	21413884–21417221 (F)	804	267	29.20	6.00	*OsPhd12* [18]
*OsPHD14*	LOC_Os02g48810	2	29871368–29875355 (F)	2064	687	71.93	5.25	*OsPhd14* [18]
*OsPHD15*	LOC_Os02g52960	2	32384631–32396094 (F)	4461	1486	164.12	7.48	
*OsPHD16*	LOC_Os03g04980	3	2409861–2413432 (F)	762	253	27.61	5.65	*OsPhd18* [18]
*OsPHD17*	LOC_Os03g19020	3	10651192–10655656 (F)	2400	799	90.27	4.54	
*OsPHD18*	LOC_Os03g19190	3	10752594–10759931 (F)	4389	1462	158.32	7.95	
*OsPHD19*	LOC_Os03g50780	3	28988380–28991862 (F)	2094	697	76.64	5.86	
*OsPHD20*	LOC_Os03g53630	3	30747354–30755128 (F)	4038	1345	146.86	6.70	*OsPhd17* [18]
*OsPHD21*	LOC_Os03g53700	3	30791616–30797021 (F)	762	253	28.77	4.66	
*OsPHD22*	LOC_Os03g58530	3	33328214–33333421 (F)	657	218	25.02	8.34	
*OsPHD23*	LOC_Os03g60390	3	34342899–34346745 (R)	744	247	27.75	5.64	*OsPhd16* [18]
*OsPHD24*	LOC_Os04g34720	4	21001722–21010737 (F)	1314	437	48.44	5.41	*OsPhd21* [18]
*OsPHD25*	LOC_Os04g35430	4	21559229–21565988 (R)	4308	1435	160.19	8.03	*OsPhd20* [18]
*OsPHD26*	LOC_Os04g36730	4	22189014–22191831 (F)	771	256	28.24	5.98	
*OsPHD27*	LOC_Os04g52020	4	30888156–30903920 (R)	2439	812	90.94	6.60	
*OsPHD28*	LOC_Os04g59510	4	35382882–35390423 (R)	1158	385	43.00	5.13	
*OsPHD29*	LOC_Os05g03430	5	1426929–1438465 (F)	2631	876	97.00	4.81	
*OsPHD30*	LOC_Os05g07040	5	3697729–3702359 (R)	777	258	28.84	5.39	
*OsPHD31*	LOC_Os05g34640	5	20542370–20547114 (F)	777	258	29.07	5.28	*OsPhd22* [18]
*OsPHD32*	LOC_Os06g01170	6	131089–138360 (F)	3078	1025	111.11	6.70	*OsPhd24* [18]
*OsPHD33*	LOC_Os06g08790	6	4393376–4401129 (F)	2445	814	91.95	8.67	
*OsPHD34*	LOC_Os06g10690	6	5575867–5584373 (F)	3420	1139	125.22	7.20	
*OsPHD35*	LOC_Os06g12400	6	6725306–6734446 (F)	2379	792	87.21	4.95	*OsHAZ1* [27]
*OsPHD36*	LOC_Os06g17280	6	10003546–10007033 (F)	2292	763	83.42	7.58	
*OsPHD37*	LOC_Os06g20410	6	11725804–11734594 (R)	1989	662	71.45	9.02	*OsPhd28* [18]
*OsPHD38*	LOC_Os06g51450	6	31161699–31169513 (R)	2400	799	87.56	9.87	
*OsPHD39*	LOC_Os06g51490	6	31184911–31187612 (R)	864	287	32.52	7.39	
*OsPHD40*	LOC_Os07g07690	7	3859755–3868364 (F)	4329	1442	156.86	8.46	
*OsPHD41*	LOC_Os07g08880	7	4606679–4614599 (F)	657	218	24.89	7.94	
*OsPHD42*	LOC_Os07g12910	7	7414568–7418059 (R)	735	244	27.37	6.39	*OsPhd34* [18]
*OsPHD43*	LOC_Os07g31450	7	18625785–18638679 (R)	6579	2192	243.66	6.37	
*OsPHD44*	LOC_Os07g41740	7	25013506–25017814 (F)	837	278	30.38	5.56	*OsPhd32* [18]
*OsPHD45*	LOC_Os07g46690	7	27902966–27912209 (R)	5022	1673	186.85	6.80	*OsPhd30* [18]
*OsPHD46*	LOC_Os07g49030	7	29353044–29356917 (R)	2361	786	88.01	4.46	*OsPhd29* [18]
*OsPHD47*	LOC_Os07g49290	7	29519451–29522145 (R)	2235	744	83.32	8.32	*OsPhd31* [18]
*OsPHD48*	LOC_Os08g01420	8	272854–276978 (R)	1692	563	62.13	6.07	*OsPhd35* [18] *OsEhd3* [28]
*OsPHD49*	LOC_Os08g32620	8	20204623–20208055 (F)	651	216	24.82	8.41	
*OsPHD50*	LOC_Os09g04890	9	2601650–2615934 (R)	3069	1022	115.53	7.48	
*OsPHD51*	LOC_Os09g21770	9	13181330–13184741 (F)	651	216	24.64	8.84	
*OsPHD52*	LOC_Os09g27620	9	16786041–16788255 (F)	2040	679	73.76	7.60	*OsMS1* [29]
*OsPHD53*	LOC_Os09g38440	9	22130437–22135245 (F)	2133	710	79.43	9.16	
*OsPHD54*	LOC_Os11g05130	11	2247706–2256169 (R)	5667	1888	206.55	5.70	*OsPhd37* [18]
*OsPHD55*	LOC_Os11g12650	11	7125124–7128857 (R)	2148	715	79.80	8.29	
*OsPHD56*	LOC_Os11g14010	11	7776646–7784194 (R)	765	254	28.51	5.16	*OsPhd40* [18]
*OsPHD57*	LOC_Os11g18770	11	10648362–10657255 (R)	3171	1056	113.99	7.91	
*OsPHD58*	LOC_Os12g24540	12	14012791–14020313 (R)	3807	1268	138.94	5.39	
*OsPHD59*	LOC_Os12g34330	12	20806080–20813363 (R)	2067	688	76.07	9.22	

## Supplementary Materials

Supplementary materials can be found at www.mdpi.com/1422-0067/18/9/2005/s1.
