# Supplementary material for: Genome-Wide Identification of the PHD-Finger Family Genes and Their Responses to Environmental Stresses in Oryza sativa L
Source: Int J Mol Sci. 2017 Sep 19;18(9):2005. doi: 10.3390/ijms18092005 (PMC5618654; doi:10.3390/ijms18092005)
Supplement: Supplementary file 1 [file ijms-18-02005-s001.docx]

Supplementary Materials: Genome-Wide Identification of the PHD-Finger Family Genes and Their Responses to Environmental Stresses in *Oryza sativa* L*.*

Mingzhe Sun, Bowei Jia, Junkai Yang, Na Cui, Yanming Zhu * and Xiaoli Sun *


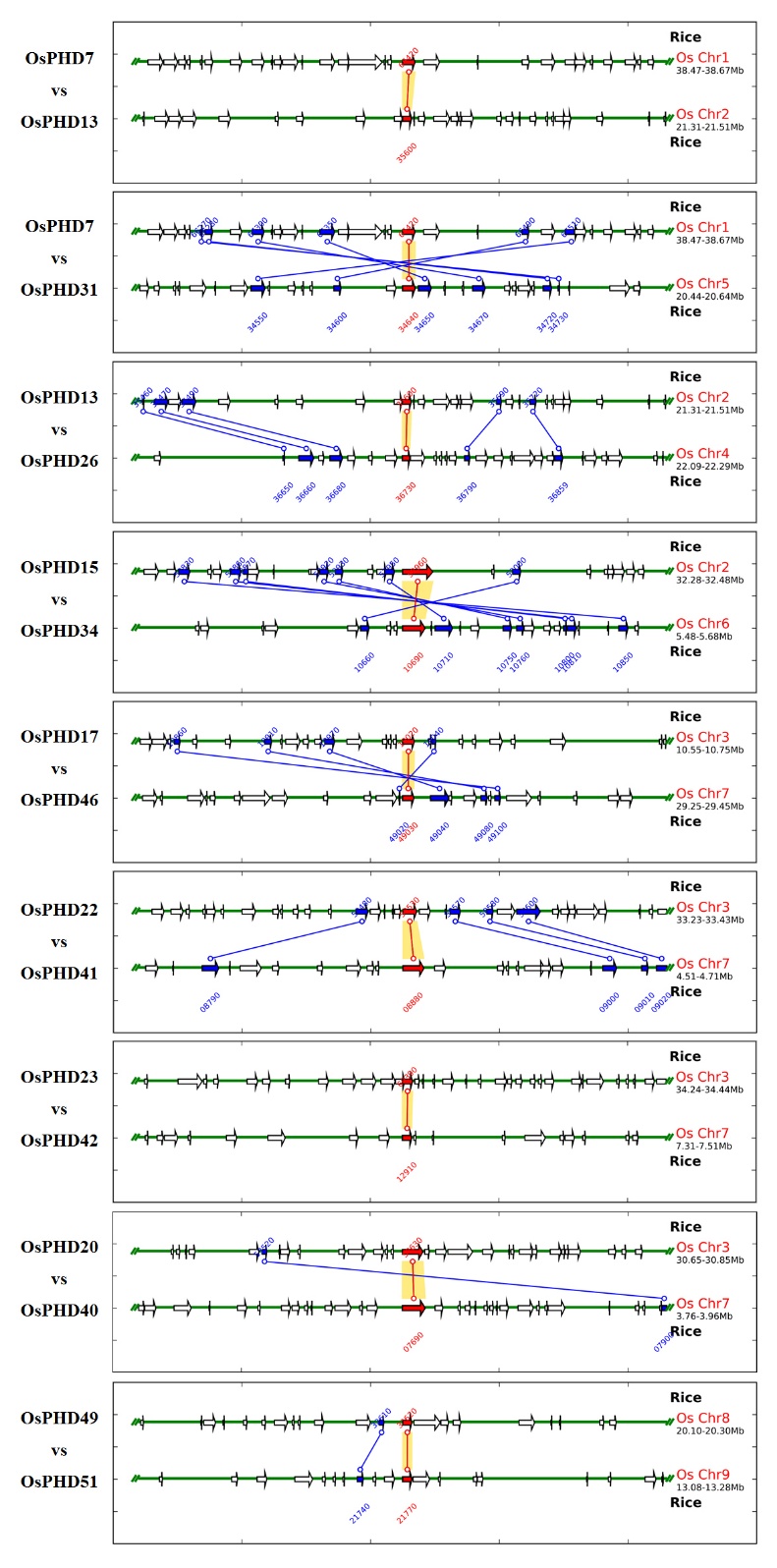


**Figure S1.** Segmental duplication analyses of the rice PHD family genes. The segmental duplication of the rice PHD family genes was obtained based on the locus search at PGDD website. The red line represents the duplication relationship between two PHDs, and the blue lines represent other gene duplication around the PHD genes.


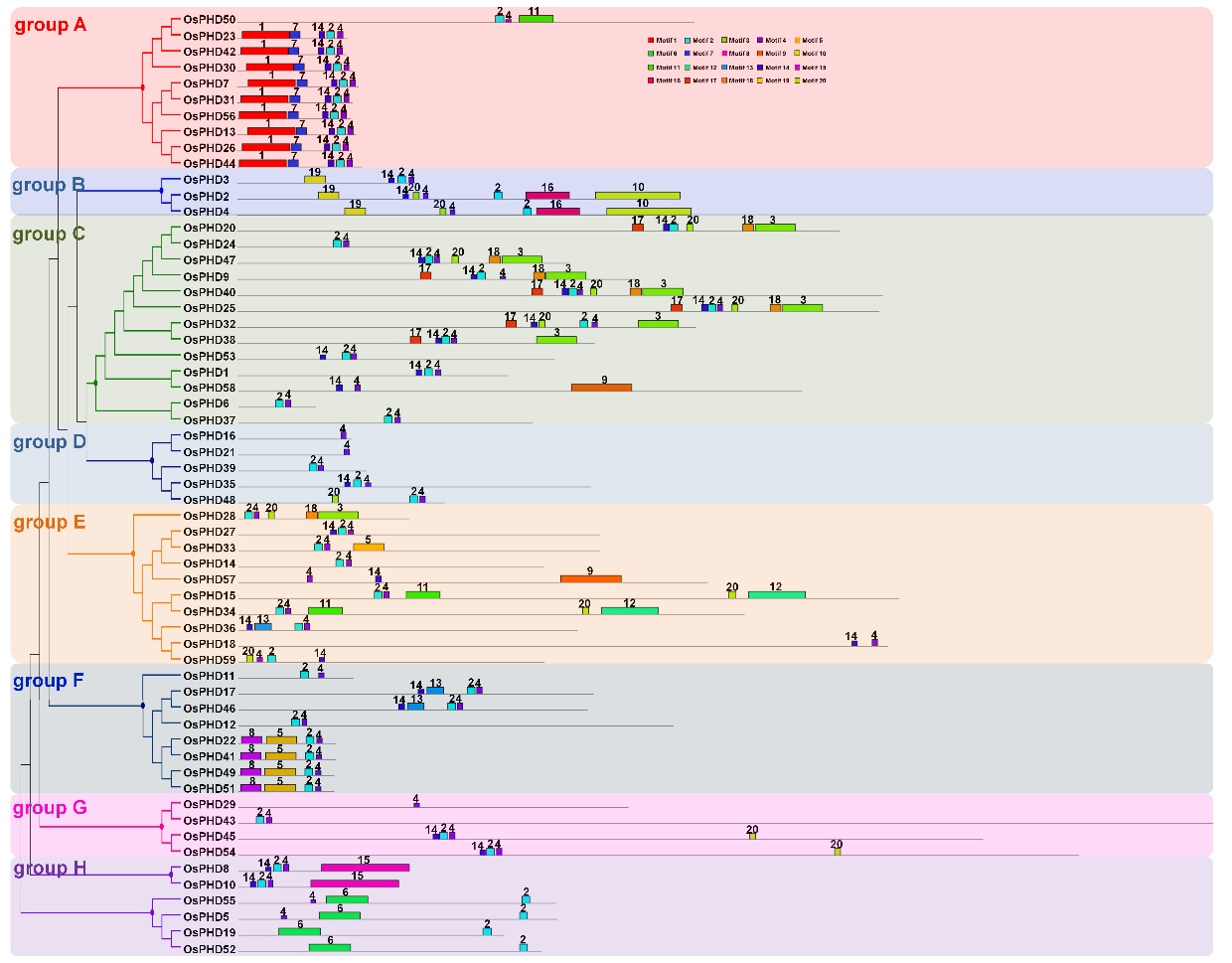


**Figure S2.** Distribution of conserved motifs within the rice PHD family. Motifs within *OsPHDs* were identified by using the online MEME program, using the following parameters: 5 ≤ optimum motif width ≤ 200; the number of motifs = 20; zero or one occurrence per sequence.


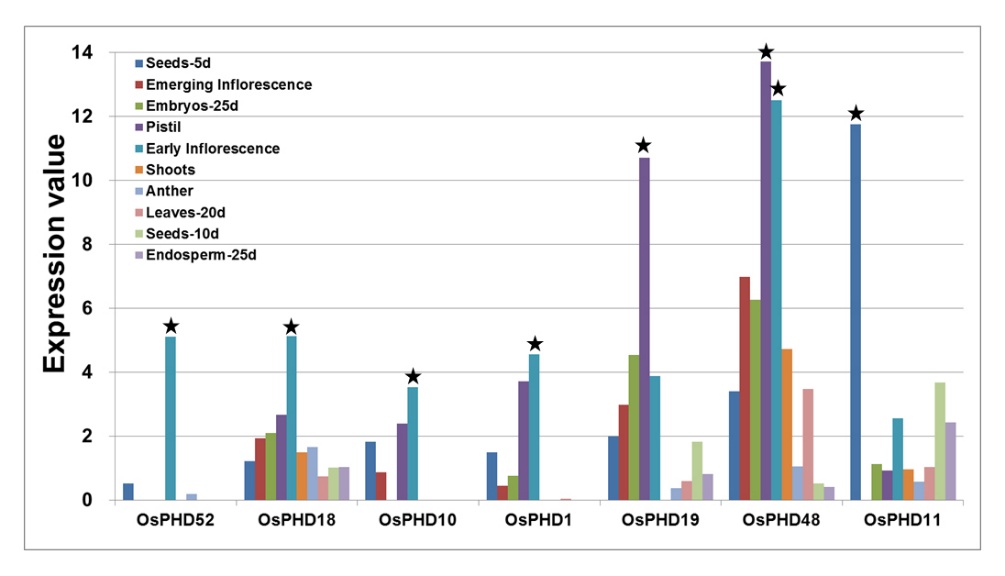


**Figure S3.** The raw data of expression values showing the tissue specific expression of Group I *OsPHDs* in Figure 6. Expression data were downloaded from the Rice Expression Profile Database. The pentagrams above the columns mark the specific tissues that *OsPHDs* expressed at the highest level.


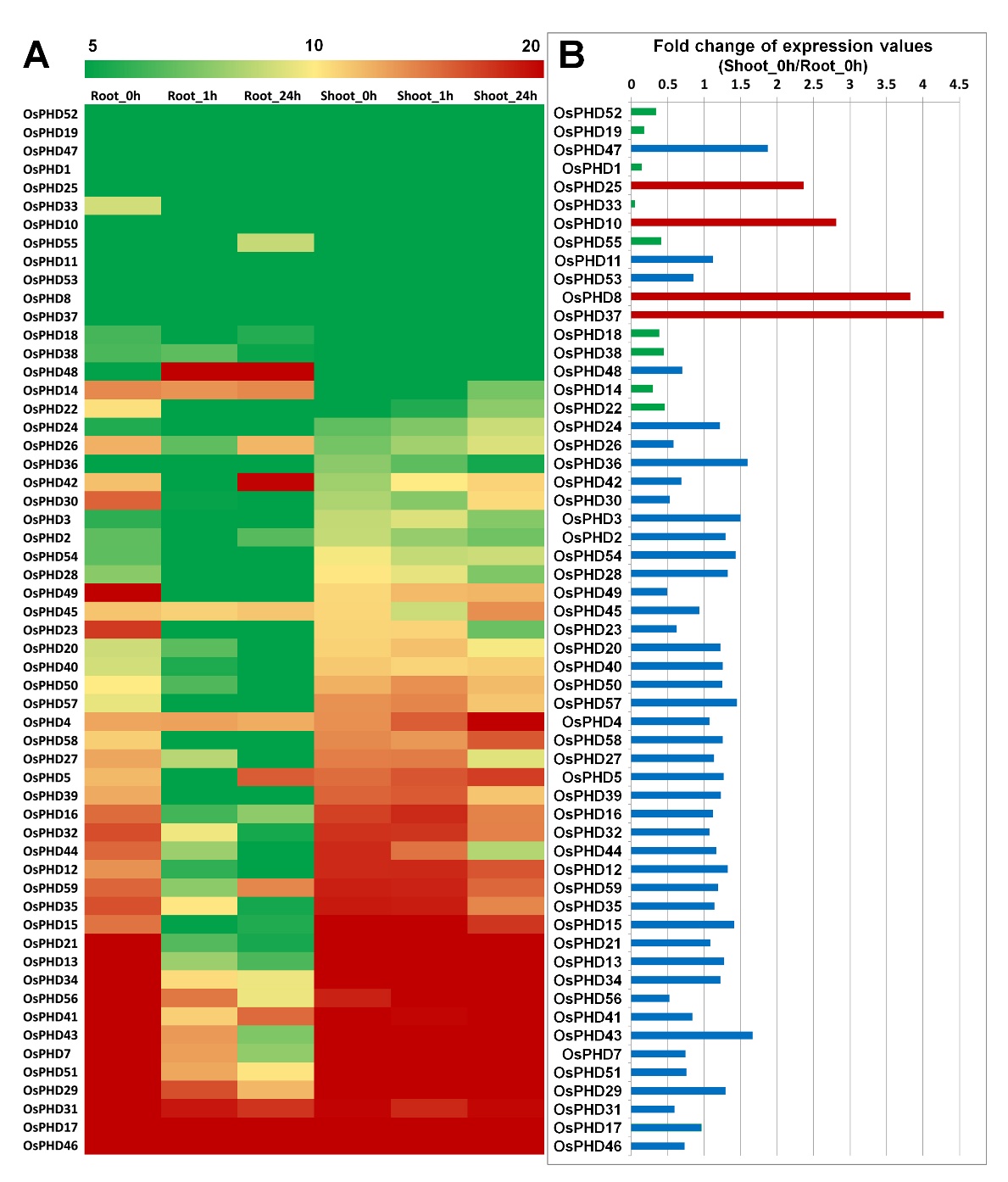


**Figure S4.** The expression of *OsPHDs* in roots and shoots in response to Cd stress. (**A**) The raw data of expression values *OsPHDs* in roots and shoots at 0, 1, and 24 h after Cd treatment. A heat map was generated with the raw expression data downloaded from the Rice Expression Profile Database. The color scale represents the expression values. Red indicates high levels and green represents low levels; (**B**) The fold change of expression values at 0 h between shoot and root. The columns mean the values of shoot_0 h/root_0 h. Green columns represent change folds < 0.5, red columns represents change folds > 2, and blue columns represent 0.5 < change folds < 2.

**Table S1.** Responses of the rice PHD family genes to diverse environmental stresses.

| **Gene name** | **Water Deficit** | **ABA** | **Cold** | **Cd** | **Responsive Stresses** |
| --- | --- | --- | --- | --- | --- |
| *OsPHD1* | − |  |  | − | Water Deficit, Cd |
| *OsPHD2* |  |  |  |  |  |
| *OsPHD3* |  | + |  | − | ABA, Cd |
| *OsPHD4* |  | + |  |  | ABA |
| *OsPHD5* | − |  | − | − | Water Deficit, Cold, Cd |
| *OsPHD6* | − |  | + |  | Water Deficit, Cold |
| *OsPHD7* | + |  |  | − | Water Deficit, Cd |
| *OsPHD8* | + |  |  | − | Water Deficit, Cd |
| *OsPHD9* |  |  |  |  |  |
| *OsPHD10* |  |  |  | + | Cd |
| *OsPHD11* |  |  |  | + | Cd |
| *OsPHD12* |  | + |  | − | ABA, Cd |
| *OsPHD13* | + |  |  | − | Water Deficit, Cd |
| *OsPHD14* | + |  |  |  | Water Deficit |
| *OsPHD15* |  | + |  | − | ABA, Cd |
| *OsPHD16* |  |  |  | − | Cd |
| *OsPHD17* | + |  | + |  | Water Deficit, Cold |
| *OsPHD18* |  | + |  |  | ABA |
| *OsPHD19* |  | + |  | − | ABA, Cd |
| *OsPHD20* |  | + |  | − | ABA, Cd |
| *OsPHD21* |  |  |  | − | Cd |
| *OsPHD22* |  |  |  | − | Cd |
| *OsPHD23* |  |  |  | − | Cd |
| *OsPHD24* | + |  |  | − | Water Deficit, Cd |
| *OsPHD25* | + |  |  | − | Water Deficit, Cd |
| *OsPHD26* |  |  |  |  |  |
| *OsPHD27* |  | + |  | − | ABA, Cd |
| *OsPHD28* |  | + |  | − | ABA, Cd |
| *OsPHD29* |  |  |  | − | Cd |
| *OsPHD30* |  |  |  | − | Cd |
| *OsPHD31* |  |  |  | − | Cd |
| *OsPHD32* |  |  |  | − | Cd |
| *OsPHD33* | + | + |  | − | Water Deficit, ABA, Cd |
| *OsPHD34* |  | + |  | − | ABA, Cd |
| *OsPHD35* |  |  |  | − | Cd |
| *OsPHD36* |  | + |  |  | ABA |
| *OsPHD37* |  |  |  |  |  |
| *OsPHD38* |  |  |  |  |  |
| *OsPHD39* |  | + |  | − | ABA, Cd |
| *OsPHD40* |  | + |  | − | ABA, Cd |
| *OsPHD41* |  |  | − | − | Cold, Cd |
| *OsPHD42* |  |  |  | − | Cd |
| *OsPHD43* |  | + |  | − | ABA, Cd |
| *OsPHD44* |  |  |  | − | Cd |
| *OsPHD45* |  |  |  |  |  |
| *OsPHD46* |  |  |  | + | Cd |
| *OsPHD47* |  | − |  | + | ABA, Cd |
| *OsPHD48* |  | + |  | + | ABA, Cd |
| *OsPHD49* |  | − |  | − | ABA, Cd |
| *OsPHD50* |  |  |  | − | Cd |
| *OsPHD51* |  |  |  | − | Cd |
| *OsPHD52* |  |  |  | − | Cd |
| *OsPHD53* |  |  |  | − | Cd |
| *OsPHD54* |  |  |  | − | Cd |
| *OsPHD55* |  |  | + | + | Cold, Cd |
| *OsPHD56* |  |  |  | − | Cd |
| *OsPHD57* |  | + |  | − | ABA, Cd |
| *OsPHD58* |  | + |  | − | ABA, Cd |
| *OsPHD59* |  | + |  | − | ABA, Cd |
| Number of Differentially Expressed Genes | 11 | 21 | 5 | 47 |  |

+: Up-regulated, Log2 fold change > 1; −: Down-regulated, Log2 fold change < −1.

**Table S2.** Gene specific primers for quantitative real-time PCR analyses.

| **Gene name** | **Primer Sequence (5' to 3')** |
| --- | --- |
| *OsPHD1* | Forward: CTCAACGAAGCAGCACGGG  Reverse: TGCAGACGGAGCAGAACCAGT |
| *OsPHD3* | Forward: GCTCGGGGATGTTGTGTGG  Reverse: CACGGAATCTGCTTGCCTTG |
| *OsPHD5* | Forward: ATCCTTGGTATGGTGAATGGGG  Reverse: TGTCTCGTTCTTCTTGCGTGCT |
| *OsPHD6* | Forward: TGTGCCCACTCCCTCTGTCC  Reverse: CCTCCTCTTCGTCTCCTTTTCCT |
| *OsPHD7* | Forward: TGAGAAAGACTGGTTGGCGTTA  Reverse: CGTTGGTTCCACAGGTCCC |
| *OsPHD8* | Forward: GAAAGCGTTCCCTTGTTATGC  Reverse: GCCACCTTCTTCCAGTGTAGC |
| *OsPHD13* | Forward: CAGTGCGACCCCGAGAAAG  Reverse: AAAGCGAGCCCCAAAGTAAAA |
| *OsPHD14* | Forward: CAGGAAACCCTCTTACAGGTCATC  Reverse: GCTCCCTTCTCACAAGCATCAC |
| *OsPHD15* | Forward: TTTGGGGGAGAGTAGTACGGTG  Reverse: CCCTTTGACAGGTTTTAGAGCG |
| *OsPHD17* | Forward: AGGGTGTGCTGGATTGCTGT  Reverse: TCCGTAGGCTGATAAACCTGATC |
| *OsPHD18* | Forward: TAGGACCACTTCAGGGGGCT  Reverse: GCACTTTGAACAAAACTGGGGA |
| *OsPHD19* | Forward: CTCTGGTTACAGTCCGTGAGTTCC  Reverse: GTGTCATCTCTGCCCCGCT |
| *OsPHD22* | Forward: GCAGAAGCGGGTGCTGGAGT  Reverse: GCGAATCATAGTGGTCAGAAAGAAAT |
| *OsPHD24* | Forward: CGAACTAAAATCAAGGCAGAGGA  Reverse: GCAACAAGACAGATGGAACGC |
| *OsPHD28* | Forward: TTGGTCCTTTCTGCCATCCC  Reverse: GCCCGTTTCCTTTCCTGCT |
| *OsPHD33* | Forward: CATCGTGAGGACCGCCAA  Reverse: GGTACAGCTCCCTACGCAAATTAT |
| *OsPHD36* | Forward: CGAGGTGTGAGATTGACGATGA  Reverse: GCCCTACTGTCCAGCCTAGTGC |
| *OsPHD41* | Forward: GTCTCCTCCCAACTCCAAACCC  Reverse: CCCTCATTAGCACACAGTCCCC |
| *OsPHD44* | Forward: TTTGCCCGCAGAAGAGGTG  Reverse: CACTCGGTGGCTTGGCTTTA |
| *OsPHD49* | Forward: GTGGGGGACTGCGTGCTGAT  Reverse: CGAAGTGGTCGGAGAGGAAGA |
| *OsPHD55* | Forward: GGGATTCAAACTTAGCAGCGG  Reverse: CACGGATTGTGGTGAGAGGGT |
| *OsPHD58* | Forward: AACAGGATGGGCTTGATATTGAA  Reverse: CCAACATCTCCACAAATGTCACA |
| *OsEf1-α* | Forward: GGAAGCCGCTGAGATGAACAA  Reverse: AAGAGCCTCAAGCAAGGTGGG |
